# Spatio-temporal evolution and influencing factors of culture and tourism integration efficiency in Shandong Province, China under high-quality development

**DOI:** 10.1371/journal.pone.0277063

**Published:** 2022-12-30

**Authors:** Fei Lu, Huaiguo Ren, Xinglong Zhai

**Affiliations:** 1 College of Culture and Tourism, Weifang University, Weifang, China; 2 Editorial Department of Journal, Weifang University, Weifang, China; Northeastern University (Shenyang China), CHINA

## Abstract

Improving culture and tourism integration efficiency is an important way to promote the high-quality development of cultural tourism. According to the inherent requirements of high-quality development, this paper constructed an evaluation indicator system for culture and tourism integration efficiency. Then, the culture and tourism integration efficiency of 16 cities in Shandong Province, China during the period from 2010 to 2019 was measured with the benevolent DEA cross-efficiency model. On the basis of exploratory spatial data analysis and dynamic spatial Durbin model, we explored the spatio-temporal evolution characteristics and influencing factors of culture and tourism integration efficiency in Shandong Province. The results show that from 2010 to 2019, the culture and tourism integration efficiency in Shandong Province has experienced three stages of "rapid growth-rapid decline-stable rise period". The spatial pattern has changed from "high in the east and low in the west" to "high in the central and low in the north and south", and regions with high integration efficiency are mainly concentrated in Jiaodong Peninsula. The level of economic development significantly promotes the culture and tourism integration efficiency in local and neighboring cities in the short and long term, while policy environment has a significant negative impact. Traffic conditions and human capital only promote the culture and tourism integration efficiency in local cities. The level of information development and openness degree only have a long-term effect on the culture and tourism integration efficiency, without short-term effect. The research results are of great significance to improve the growth quality and sustainable development of cultural tourism in Shandong Province. Our work could provide a scientific basis for maximizing the allocation benefits of cultural and tourism resources in similar regions in the world.

## Introduction

Since China’s 13th Five-Year Plan, the cultural tourism industry in Shandong Province has maintained a rapid growth, and the added value of the cultural industry and the total tourism consumption have been at the forefront of China’s provinces for many years. Shandong Province’s "Culture and Tourism Integration Development Plan (2020–2025) " and "14th Five-Year Plan (2021–2025) for National Economic and Social Development and Vision 2035" all take "promoting the deep integration of culture and tourism" as a key task. However, at present, there are some outstanding problems in cultural tourism in Shandong Province, such as large but not strong industry and low degree of intensification. In the context of high-quality development, the cultural tourism industry is facing the urgent requirement of changing from the speed of development to the quality of development. In order to build a strong cultural tourism province, Shandong possessed a great number of advantages. Based on the rich culture and tourism resources, giving full play to the advantages of location, economy and ecological environment, improving the quality of integration of cultural industry and tourism industry, and creating a number of competitive cultural tourism demonstration zones are the realistic choices for Shandong. Culture and tourism integration efficiency reflects the internal relationship and ratio between the input and output of cultural tourism economic activities, and is one of the indicators to measure the quality of cultural industry and tourism industry integration in a region. By improving culture and tourism integration efficiency, we can optimize the allocation of resources for the development of cultural tourism industry, improve the speed and quality of integration of cultural industry and tourism industry, and then enhance the overall strength of cultural tourism industry. Therefore, it is of great theoretical and strategic significance to apply scientific and accurate methods to measure the culture and tourism integration efficiency in Shandong Province, reveal its temporal and spatial characteristics and influencing factors, and explore the optimization direction of in-depth integration of culture and tourism on this basis.

The study of culture and tourism integration efficiency originated from the discussion of the relationship between culture and tourism by scholars. As early as the 16th century Grand Tour, cultural experience has been regarded as one of the main purposes of tourism [1]. After entering the 20th century, cultural experience is no longer regarded as the purpose of tourism, but as an interdependent relationship [2]. In the 1970s, after Robert Macintosh first put forward the concept of "tourism culture", the conceptual integration system of culture and tourism was really established [3]. As the concept of culture getting closer to daily life, the normative definition of "cultural tourism" has been extended by relevant industry organizations, such as the United Nations World Tourism Organization (UNWTO) and the European Association for Tourism and Leisure Education (ATLAS) [4]. At the same time, the applied research on integration of culture and tourism at the practical level has attracted wide attention. In 1982, the Ministry of Art of the United States made clear the mutual benefit of culture and tourism. Compared with other forms of tourism, cultural tourism is more popular, and cultural industry and tourism organizations are more and more active in promoting the relationship between the two sides [5]. In 2018, the World Tourism Organization emphasized the importance of synergies between cultural industries and tourism institutions in "Tourism and Culture Synergies". On the whole, the discussion of the relationship between culture and tourism has gradually extended to the aspects of interdependence and synergy between cultural industry and tourism industry [6]. Specifically, the research topics of culture and tourism integration mainly include the types of business integration, the mutual influence of integration and the industrialization of integration.

Cultural heritage tourism research has always been a hot topic in the type of business integration. Cultural industry and tourism industry clusters based on cultural heritage are important elements which enhance regional competitiveness [7]. The development of tourism in cultural heritage tourism destinations promotes the process of cultural activation, makes culture more experiential. The development is conducive to the inheritance and dissemination of traditional culture [8]. In recent years, the relationship between cultural creativity and tourism has developed rapidly, and its fundamental driving force is the market integration caused by cultural demand [9]. Cultural creativity is used to change traditional cultural tourism from tangible heritage to more intangible culture [10], such as tourism performing arts, film and television tourism, etc. In terms of the mutual influence of integration, Qu et al. believed that whether tourism has a positive impact on national culture depends on the recognition of cultural citizenship [11]. Ma et al. analyzed the sustainable livelihoods of households a typical rural tourist site in Wuyuan County, Jiangxi Province, and found that the development of rural tourism with material cultural resources and intangible cultural heritage promoted the transformation of farmers’ livelihood strategies, but the development of rural tourism made cultural protection a potential source of farmers’ livelihood vulnerability [12]. Su et al. established an interdisciplinary composite analytical framework from the perspective of efficiency evaluation, and showed that after the intervention of cultural industry, the technical efficiency of tourism industry has been significantly improved, and there is a synergistic effect between culture and tourism [13]. With the increasingly close integration of tourism and emerging cultural formats, the influence of fashion culture or pop culture on tourist destinations has gradually attracted scholars’ attention. Seo and Kim pointed out that "Hallyu" product implantation can become a powerful marketing strategy in the tourism industry [14]. Whang et al. believed that popular culture played an important role in guiding young people’s choice of tourist destinations and their tourism behavior [15]. However, some scholars pointed out that local popular culture should be treated with caution in tourism development. Popular culture may pose a certain threat to the authenticity of tourism destination culture. Although popular culture may become a new attraction factor for tourism destinations, it was not always advisable for tourism destinations to use popular culture to develop tourism products [16]. In terms of integration industrialization, the mechanism [17], motive force [18], path [19] and mode [20] of integration of cultural industry and tourism industry are discussed from the perspective of industrial integration theory. On this basis, the integration level of cultural industry and tourism is quantitatively evaluated. Most of the existing studies have established the index system and model of the coupling system of cultural industry and tourism industry, used the coupling function method to study the relationship between coupling degree and system synergy [21–23], explored the regional differences and spatial differentiation rules of the coupling relationship between cultural industry and tourism industry based on panel data [24], and revealed the specific performance of different integration stages from the mechanism level [25, 26].

Through the combing of the existing literature, the current academic research on the integration of culture and tourism has a wide range, but there are still weak links. First of all, the current research mainly focuses on the coupling and coordination between cultural industry and tourism industry. Although it can reveal the state of coordinated development of cultural industry and tourism industry, it is impossible to judge the quality of integration, whether the operation of integration is normal and whether the practice is reasonable, thus affecting the effectiveness of policy recommendations. From the perspective of the integration of cultural industry and tourism industry, Liebowitz and Margolis believed that the path dependence caused by low technical efficiency was the main obstacle to the improvement of industrial quality [27]. Huang and Xu proposed that industrial efficiency reform can change the path dependence of cultural and tourism industry integration, thereby improving the level of culture and tourism integration [28]. Thus, efficiency development is the key to realize the supply-side reform of cultural industry and tourism industry, which is conducive to constantly changing and optimizing the efficiency of resource allocation of industrial integration. Therefore, it is urgent to further study the integration of culture and tourism from the perspective of efficiency. Secondly, in the selection of cases, mainly at the national level, and there is a lack of quantitative research on provincial scale. Shandong Province has two national cultural and tourism consumption demonstration cities, Jinan and Qingdao, and the demonstration effect of culture and tourism integration is obvious, so it is particularly necessary to carry out in-depth analysis of culture and tourism integration efficiency. In addition, although the current research on integration of culture and tourism involves the analysis of integration mechanism and spatial differentiation, it ignores the influence of spatial factors, which easily leads to systematic model setting bias. Based on this, this paper took 16 cities in Shandong Province, China as the research object, starting from the requirements of high-quality development in the new era, scientifically constructed an evaluation index system for culture and tourism integration efficiency, and used the benevolent DEA cross-efficiency model to measure the culture and tourism integration efficiency of 16 cities in Shandong Province from 2010 to 2019. It applied the spatial autocorrelation method to analyze the spatio-temporal evolution characteristics of culture and tourism integration efficiency in Shandong Province. Then, we utilized the dynamic spatial Durbin panel model to explore the influencing factors of spatio-temporal differentiation of culture and tourism integration efficiency.

Compared with the previous studies, the marginal academic contributions of this paper are as follows: Firstly, in terms of research methods, the benevolent DEA cross-efficiency model combining self-and peer-appraisal is used to solve the inherent problem that the traditional DEA model can not effectively distinguish the advantages and disadvantages of each unit based on peer-appraisal, which is conducive to scientifically measuring the culture and tourism integration efficiency of 16 cities in Shandong Province. Secondly, in terms of research content, the essential connotation and goal of high-quality development, innovation-driven development and green sustainable development, are incorporated into the evaluation index system of culture and tourism integration efficiency, highlighting the realistic requirements of the new development concept. Considering the dynamic sustainability of culture and tourism integration efficiency, this paper uses the dynamic spatial Durbin model to analyze the direct and indirect effects of different factors on culture and tourism integration efficiency, and more comprehensively evaluates the factors affecting culture and tourism integration efficiency in Shandong Province. Thirdly, in terms of application value, through an all-round and multi-angle investigation of the culture and tourism integration efficiency in 16 cities of Shandong Province under high-quality development, it not only provides theoretical basis and practical support for the resource element allocation bottleneck and regional coordination policy problems of culture and tourism integration in Shandong Province and other regions under similar conditions, but also provides decision-making reference for promoting the integration and development of culture and tourism in a wider scope, deeper level and higher level during the 14th Five-Year Plan period.

## Materials and methods

### Benevolent DEA cross-efficiency model

The traditional DEA model is based on the idea of self-appraisal, which often leads to multiple DMUs being effective at the same time, and can not further distinguish the efficiency differences of each effective DMU. In order to solve this problem, Sexton et al. proposed the DEA cross-efficiency model, which introduced the peer-appraisal mechanism to make each DMU accept the evaluation of other DMUs, so as to alleviate or eliminate the drawbacks of the self-appraisal system [29]. However, it ignores that the optimal weight combination for solving the cross-efficiency value is not unique. So as to further improve the DEA cross-efficiency model, Doyle introduced the quadratic objective model, and then constructed the aggressive and benevolent cross-efficiency models [30]. On the premise that the optimal self-evaluation efficiency value of the evaluated DMU remains unchanged, minimize and maximize the average efficiency values of the other DMUs to determine a set of optimal weights. Considering that the integration of cultural industry and tourism industry is beneficial to the development of both sides, this paper chooses the benevolent DEA cross-efficiency model.

Suppose there are *n* DMUs, denote as *DMU*_*j*_ (*j* = 1,2,…*n*). Each DMU has m inputs, denotes as *x*_*i*_ (*i* = 1,2,…*m*), and *q* outputs, denotes as *y*_*r*_ (*r* = 1,2,…*q*). Then, the self-assessment efficiency value *E*_*dd*_ of *DMU*_*d*_ under the CCR model is:

$$\begin{array}{c} E_{dd}=\max\sum_{r=1}^{q}\mu_{r}{}_{d}y_{rd}\\ s.t.\left\{ \begin{array}{l} \sum_{r=1}^{q}\mu_{r}{}_{d}y_{rj}-\sum_{i=1}^{m}\nu_{i}{}_{d}x_{ij}\leq 0\\ \sum_{i=1}^{m}\nu_{id}x_{id}=1\\ \;\nu_{id}\geq 0;\;\mu_{rd}\geq 0\\ \;i=1,2,\cdots ,m;\;r=1,2,\cdots ,q;\;j=,2,\cdots n \end{array}\right. \end{array}$$ (1)

The cross efficiency value *E*_*dj*_ (*d* = 1,2,…*n*) of *DMU*_*j*_ is calculated by using the optimal weight combination of the remaining *n-1* DMUs, the efficiency value *E*_*j*_ obtained after averaging:

$$E_{j}=\frac{1}{n}\frac{\sum_{d=1}^{n}\sum_{r=1}^{q}\mu_{rd}^{*}y_{rj}}{\sum_{d=1}^{n}\sum_{i=1}^{m}\nu_{id}^{*}x_{ij}}$$ (2)

The benevolent DEA cross-efficiency model of *DMU*_*j*_ relative to *DMU*_*d*_ is:

$$\begin{array}{l} \;\max\sum_{r=1}^{q}\mu_{r}{}_{d}(\sum_{j=1,j\neq d}^{n}y_{rj})\\ s.t.\sum_{r=1}^{q}\mu_{r}{}_{d}y_{rj}-\sum_{r=1}^{m}\nu_{i}{}_{d}x_{ij}\leq 0\\ \;\sum_{i=1}^{m}\nu_{id}(\sum_{j=1,j\neq d}^{n}x_{ij})=1\\ \;\sum_{r=1}^{q}\mu_{r}{}_{d}y_{rd}-E_{dd}\sum_{r=1}^{m}\nu_{i}{}_{d}x_{id}\\ \;\nu_{id}\geq 0;\;\mu_{rd}\geq 0\\ \;i=1,2,\cdots ,m;\;r=1,2,\cdots ,q;\;j=1,2,\cdots n \end{array}$$ (3)

### Exploratory Spatial Data Analysis method (ESDA)

Exploratory spatial data analysis is an important field of spatial econometrics, which explains spatial dependence, spatial correlation or spatial autocorrelation related to spatial location. It also explores spatial agglomeration and spatial anomalies by describing and visualizing the spatial distribution pattern of things or phenomena. In this study, by calculating Global Moran’s I and Local Moran’s I, we analyzed the spatial characteristics of culture and tourism integration efficiency of 16 cities in Shandong Province on global and local dimensions, respectively.

#### Global spatial auto-correlation

Global spatial auto-correlation is used to describe the overall distribution of culture and tourism integration efficiency, so as to judge whether it has the characteristics of agglomeration or dispersion in space. The commonly used test statistic is the global Moran’s index [31], denoted as *I*, which can be written as:

$$I=\frac{n\sum_{i=1}^{n}\sum_{j=1}^{n}W_{ij}(x_{i}-\bar{x})(x_{j}-\bar{x})}{\sum_{i=1}^{n}\sum_{j=1}^{n}W_{ij}\sum_{i=1}^{n}{\left(x_{i}-\bar{x}\right)}^{2}}$$ (4)

In this expression, *n* denotes the number of regions. x_i_ and x_j_ denote the integration efficiency of culture and tourism in regions *i* and *j*. *W*_*ij*_ denotes the spatial weight matrix. Considering the deficiency of geographical distance weight matrix in revealing the agglomeration of economic phenomena and the unbalanced state of adjacency matrix caused by regional area gap, this paper chooses the K-nearest neighbor weight matrix of distance and adjacency combination.

#### Local spatial auto-correlation

Local spatial auto-correlation is used to describe the correlation degree of culture and tourism integration efficiency between a region and its neighboring regions, so as to judge the agglomeration or dispersion characteristics between local regions. The commonly used test statistic is the local Moran’s index [32], denoted as *I*_*i*_, which can be calculated as:

$$I_{i}=\frac{(x_{i}-\bar{x})\sum_{j}^{}W_{ij}(x_{j}-\bar{x})}{\sum_{j=1,j\neq i}^{n}{\left(x_{j}^{}-\bar{x}\right)}^{2}/\left(n-1\right)}$$ (5)

### Dynamic Spatial Durbin Panel Model (DSDM)

Compared with the traditional regression model, spatial econometric model can effectively solve the complex spatial correlation and dependence in the regression process. Spatial Durbin model (SDM) takes into account not only the spatial lag terms of the explanatory variables, but also the spatial lag terms of the explained variables, which can more accurately describe the spatial relationship and interaction of various economic activities [33]. In addition, considering the economic inertia, the first-order lag term of explained variable is introduced into the spatial Durbin model to construct the dynamic spatial Durbin panel model [34], and the formula is expressed as follows:

$$y_{it}=\partial_{0}+\partial_{1}y_{i,t-1}+\eta \sum_{j=1}^{n}W_{ij}y_{j,t-1}+\rho \sum_{j=1}^{n}W_{ij}y_{jt}+\partial_{2}X_{it}+\partial_{3}\sum_{j=1}^{n}W_{ij}X_{jt}+\mu_{i}+\xi_{t}+\varepsilon_{it}$$ (6)

In this expression, *y*_*it*_ denotes the explained variable at time *t* in region i, which is the culture and tourism integration efficiency for this paper. *W*_*ij*_ denotes the spatial weight matrix. α_1_ denotes the time lag coefficient. *ρ* denotes the spatial autoregressive coefficient. *η* denotes the spatio-temporal lag coefficient. *X* denotes the explanatory variable. *μ*_*i*_ denotes the regional fixed effect. *ξ*_*t*_ represents the time fixed effect. *ε*_*it*_ denotes the random error term.

## Indicators and data

### Evaluation indicators system of culture and tourism integration efficiency

Culture and tourism integration is a superimposed evolution process of mutual influence and interaction between cultural industry and tourism industry, which is essentially a phenomenon of convergence. According to the convergence model and its assumptions [35], culture and tourism integration is a process or process state in which the two interact and promote to achieve technical efficiency. Through this process or process state, we can realize the promotion of tourism industry by cultural industry and the promotion of cultural industry by tourism industry. Among them, technical efficiency refers to the ability to minimize the input resources of cultural industry under the given output conditions of tourism industry (that is, the promotion efficiency of cultural industry to tourism industry), or the ability to minimize the input resources of tourism industry under the given output conditions of cultural industry (that is, the promotion efficiency of tourism industry to cultural industry). Based on the research results of industrial integration efficiency [36, 37], this paper defines the culture and tourism integration efficiency as the smaller value of the promotion efficiency of cultural industry to tourism industry and the promotion efficiency of tourism industry to cultural industry. Summarize the existing research results [38–40], on the basis of data availability, the data that can maximize the input and output of the urban unit cultural tourism industry under high-quality development are selected and included in the benevolent DEA cross-efficiency model to make the evaluation results more realistic and accurate.

Input indicators. In the selection of input variables, it reflects the input generated by investment in the development of cultural tourism industry from the perspective of capital and labor. The number of direct people working in cultural tourism industry is the most intuitive criterion for reflecting the elements of labor, but the relevant statistical yearbook does not have detailed data combing. So the number of people working in cultural, sports and entertainment industries is selected instead of the labor input of cultural industry, and the number of people working in accommodation and catering industry is selected to replace the labor input of tourism industry. The investment of capital factors mainly includes the infrastructure and services of cultural tourism industry, but most cities lack statistical data on this aspect. Therefore, the indicators are selected from the perspective of the attractiveness of cultural tourism industry. Cultural industry includes the number of public libraries, art performance venues, cultural centers and museums. Tourism industry includes the number of 4A-level and above tourist attractions, four-star and above hotels, and travel agencies. High-quality development requires the implementation of the new development concept of "innovation, coordination, green, openness and sharing", whose essential connotation and goal are high efficiency, fairness and green sustainable development [41]. Therefore, the measurement of culture and tourism integration efficiency under high-quality development should highlight two aspects: First, high efficiency to promote innovation-driven development. Combining Romer’s endogenous growth theory and the realistic requirements of high-quality development driven by innovation, this paper includes technological progress into the input indicators [42], and expresses the technological progress of cultural industry and tourism industry by the number of cultural and tourism social science fund projects respectively. Second, green and sustainable development to achieve integrated development of low energy consumption and pollution. When measuring the culture and tourism integration efficiency, we need to treat resources and environmental factors correctly, so as to more profoundly reflect the realistic requirements of high-quality development in the new era. On this basis, the energy consumption is included in the input indicators, and the energy consumption per unit of tourism industry revenue is selected to represent the energy consumption of tourism industry. The energy consumption per unit of the added value of cultural industry is selected to represent the energy consumption of cultural industry. Pollutant emissions are the price which needs to be paid for the output of cultural tourism industry. Drawing on relevant research results [43, 44], this research treats it as a cost input variable for the cost of environmental degradation. Measuring the environmental pollution of cultural industry by the discharge amount of "three wastes" in cultural industry, and measuring the environmental pollution of tourism industry by the discharge amount of "three wastes" in tourism industry. Due to the lack of related statistics of the energy consumption and environmental pollution related indicators of cultural tourism in various cities, we selected the ratio of added value of cultural industry to GDP and the ratio of tourism revenue to GDP for conversion.

Output indicators. The added value of cultural industry and tourism revenue are the most direct products of the city’s cultural tourism industry production activities. These two indicators are also selected as the output of cultural industry and tourism industry, and the tertiary industry price index is used to deflate. Based on the DEA model’s requirements for the number of DMU and the principle of "orderly" of input-output indicators, this paper constructs an evaluation indicator system of culture and tourism integration efficiency under high-quality development (Table 1).

**Table 1:** Index system for measuring the integration efficiency of culture and tourism under high quality development.

Measure content	Indicator type	Level indicators	Secondary indicators
**Promotion efficiency of cultural industry to tourism industry**	Input indicators	Labor input	Number of people working in culture, sports and entertainment
		Capital input	Number of public libraries, art performance venues, cultural centers and museums
		Technological progress	Number of cultural and social science fund projects
		Energy input	Energy consumption per unit of cultural industry income
		environmental pollution cost	Discharge amount of "three wastes" in cultural industry
	Output indicators	Efficiency output	Revenue from tourism
**Promotion efficiency of tourism industry to cultural industry**	Input indicators	Labor input	Number of people working in the accommodation and catering industry
		Capital input	Number of 4A-level and above tourist attractions, four-star and above hotels, and travel agencies.
		Technological progress	Number of tourism social science fund projects
		Energy input	Energy consumption per unit of tourism industry income
		Environmental pollution cost	Discharge amount of "three wastes" in tourisml industry
	Output indicators	Efficiency output	Added value of cultural industry

### Dynamic spatial Durbin panel model indicators

The spatio-temporal evolution of culture and tourism integration efficiency [7] is the result under the action of multiple factors. Moreover, various influencing factors differ in direction and strength in varying degrees. It is necessary to further examine the spatial-temporal heterogeneity of various influencing factors. With reference to existing studies [45–47], and combined with the actual situation of Shandong Province, economic development level (*ECON*), human capital (*HC*), policy environment (*PE*), information development level (*INFO*), traffic condition (*TC*) and openness degree (*OD*) were selected as the independent variables for establishing the dynamic spatial Durbin panel model so as to conclude the factors that affect the difference of culture and tourism integration efficiency among different regions.

Economic development level is inextricably linked to the development of various industries that can provides the economic basis for the integration of cultural tourism industry, and reflect the actual effect of economic growth on cultural tourism consumption. In this study, GDP per capita was used for measuring the economic development level. High-quality human capital is more likely to bring knowledge and technology spillover, which is conducive to improving the allocation efficiency of production factors in cultural tourism industry. Human capital can be characterized by the per capita wage of cultural tourism employees. Policy environment is not only an important means of support and investment for the integration of culture and tourism industry, but also an important mechanism to promote the development and efficiency of cultural tourism industry. To be specific, the environmental cost can be calculated by cultural tourism, sports and media expenditure. Information development level is the bridge of spatial agglomeration and radiation of production factors of cultural tourism industry and can be calculated by the total amount of post and telecommunications business. Convenient transport facilities and development traffic networks are the premise of both prosperity and development of cultural tourism industry since they can increase the convenience of cultural tourism activities. We mainly use the density of roads to measure the traffic condition. Openness degree is tightly related to expanding cultural tourism exchanges and cooperation and forming a new situation of co-construction and sharing. Therefore, we analyze openness degree using the ratio of the total volume of imports and exports to regional GDP.

In order to avoid heteroscedasticity, except for the proportional variable, the remaining variables are treated logarithmically.

### Data source and processing

The selected sample data of 16 cities in Shandong Province, China from 2010 to 2019. All data were sourced from "Shandong Statistical Yearbook", "China Urban Statistical Yearbook", the statistical yearbooks of various cities and the statistical bulletin of national economic and social development over the years. The number of cultural and tourism social science fund projects comed from Shandong Social Science Network and Shangdong Social Science Planning Network, which were searched by keywords such as "culture, media, tourism, leisure" and summarized according to the classification of the city where they were located. Considering the continuity of the data, the data of Laiwu City before 2019 was merged into Jinan City. For the missing data of the year, the interpolation method is used to supplement.

## Results

### Temporal series characteristics of culture and tourism integration efficiency

According to Formulas (1), (2) and (3), Matlab R2011b software is used to calculate the culture and tourism integration efficiency of 16 cities in Shandong Province from 2010 to 2019, as shown in Table 2.

**Table 2:** Value of culture and tourism integration efficiency of 16 cities in Shandong Province from 2010 to 2019.

City	2010	2012	2014	2015	2017	2018	2019	Mean value
**Jinan**	0.406	0.265	0.442	0.420	0.559	0.680	0.656	0.514
**Qingdao**	0.784	0.821	0.824	0.743	0.787	0.824	0.764	0.783
**Zibo**	0.735	0.379	0.471	0.445	0.433	0.471	0.501	0.501
**Zaozhuang**	0.280	0.312	0.340	0.335	0.315	0.333	0.474	0.355
**Dongying**	0.273	0.338	0.303	0.398	0.361	0.365	0.408	0.399
**Yantai**	0.813	0.733	0.203	0.731	0.768	0.841	0.696	0.707
**Weifang**	0.622	0.617	0.591	0.625	0.706	0.791	0.678	0.638
**Jining**	0.387	0.463	0.544	0.496	0.541	0.597	0.542	0.503
**Tai’an**	0.569	0.332	0.322	0.263	0.485	0.544	0.287	0.394
**Weihai**	0.496	0.329	0.481	0.476	0.499	0.652	0.391	0.475
**Rizhao**	0.289	0.350	0.133	0.204	0.296	0.411	0.257	0.286
**Linyi**	0.640	0.642	0.367	0.463	0.593	0.613	0.492	0.538
**Dezhou**	0.193	0.331	0.292	0.271	0.214	0.212	0.259	0.278
**Liaocheng**	0.203	0.252	0.212	0.240	0.246	0.287	0.395	0.276
**Binzhou**	0.305	0.292	0.180	0.317	0.263	0.246	0.356	0.317
**Heze**	0.139	0.179	0.188	0.169	0.156	0.190	0.314	0.201

**Fig 1:**
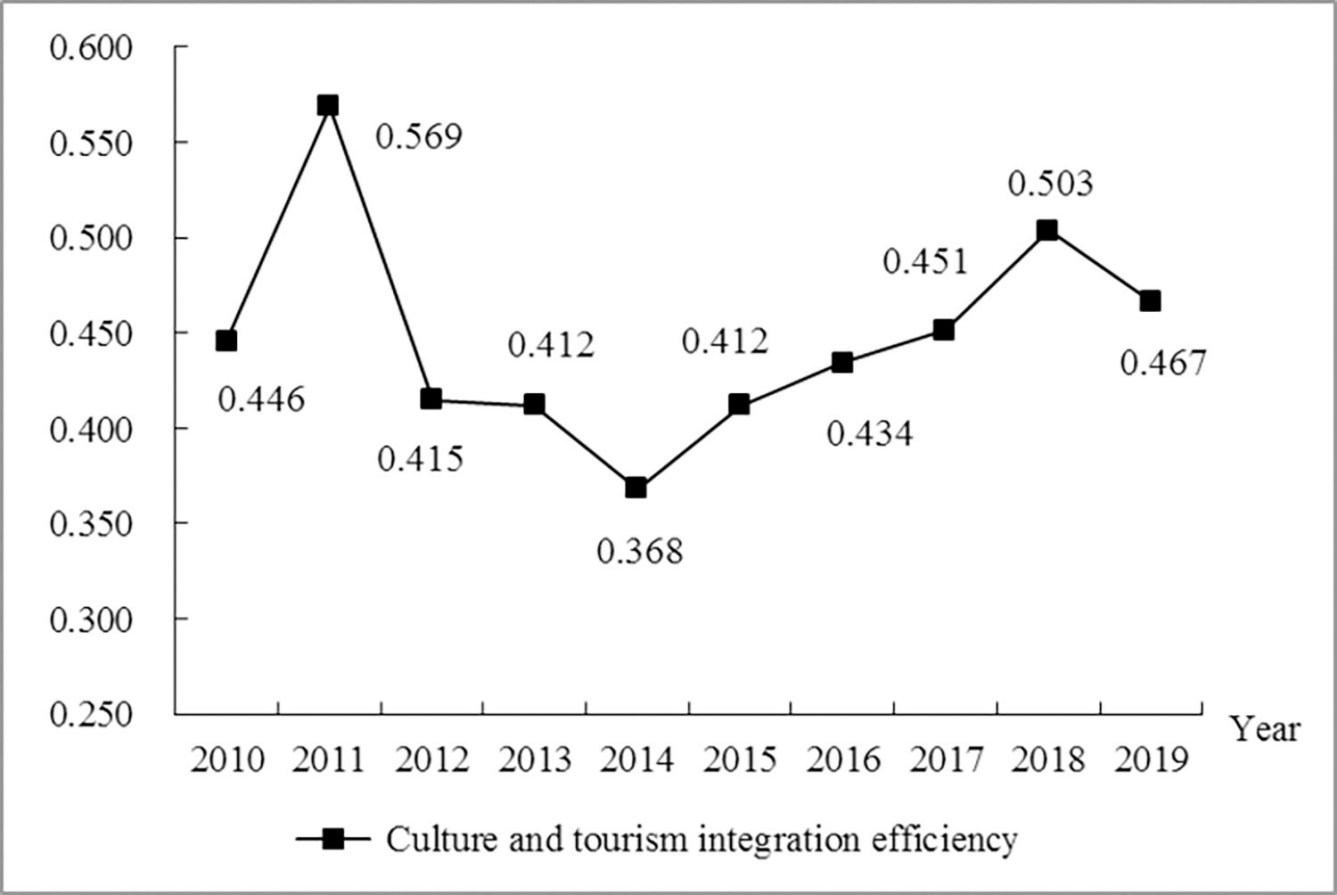
Temporal series characteristics of culture and tourism integration efficiency in Shandong Province from 2010 to 2019.

From the perspective of temporal series characteristics, the change of culture and tourism integration efficiency in Shandong Province shows a strong stage. The overall change can be divided into three stages (Fig 1): The first stage (2010–2011) is a period of rapid growth. The culture and tourism integration efficiency in this stage has increased from 0.446 to 0.569, the increase rate reached 27.57%. Affected by the stability of the external environment and favorable economic development situation, cultural tourism industry in Shandong Province has grown rapidly. Additionally, the development strategy of expanding domestic demand and stimulating consumption has accelerated the flow of resource elements in cultural tourism industry. In 2008 and 2009, Shandong Province successively issued a series of documents such as "Opinions on Promoting the Great Development and Prosperity of Culture" and "Opinions on Further Promoting the Sound and Rapid Development of Tourism Industry", which clarified the specific direction of culture and tourism integration efficiency, and these measures have greatly improved the culture and tourism integration efficiency. The second stage (2011–2014) is a period of rapid decline. The culture and tourism integration efficiency in this stage has dropped from 0.569 to 0.368, with an average annual decline of 11.78%. This results is due to the fact that although the cultural tourism industry has maintained rapid development and investment in labor and capital has expanded during this period, blind investment in cultural tourism projects, imperfect integration policy system and other problems are common, and there are insufficient opening up to the outside world and unreasonable allocation of resource elements in the cultural tourism industry between cities. The third stage (2014–2019) is a stable rise period. The culture and tourism integration efficiency in this stage has increased from 0.368 to 0.467 in 2019, with an average annual increase of 5.38%, which is due to the great progress of Shandong Province in the construction of public cultural tourism facilities and innovation-driven development at this stage. At the same time, driven by the merger of cultural and tourism institutions and the supply-side structural reform of the cultural tourism industry, new concepts and new business formats such as "cultural tourism +" and "+ cultural tourism" continue to emerge. The resource elements of cultural tourism industry can flow more freely and rationally allocate, which has been brought about by breakthroughs in the reform of cultural tourism industry system and management innovation. Although there is a decline in 2019, the overall trend is on the rise. The reason for the significant decline in culture and tourism integration efficiency may be that with the slowdown of GDP growth in Shandong Province in 2019, the tourism industry has been significantly negatively driven, which leads to the decline in culture and tourism integration efficiency.

### Spatial evolution characteristics of culture and tourism integration efficiency

#### Spatial pattern evolution characteristics

We selected the years of 2010, 2015 and 2019 as the time points to depict the spatial pattern and evolution of culture and tourism integration efficiency at the city level of Shandong Province. With the support of ArcGIS10.2 software, the culture and tourism integration efficiency in Shandong Province is divided into four levels from low to high. Thus 0<integration efficiency ≤0.250 denotes a low efficiency region, 0.251<integration efficiency≤0.500 denotes a medium efficiency region, 0.501<integration efficiency≤0.750 denotes a relatively high efficiency region, 0.751<integration efficiency≤1 denotes a high efficiency region.

The spatial distribution of culture and tourism integration efficiency in Shandong Province showed a spatial pattern of "high in the east and low in the west" in 2010. The high integration efficiency regions include two cities, Qingdao and Yantai, the highest value is Yantai, and the culture and tourism integration efficiency is 0.813. There are eleven cities in the relatively high integration efficiency and medium integration efficiency regions. All of which are distributed in the eastern and central regions, among which the medium integration efficiency regions including seven cities, accounting for 43.75% of the total, it is the most important distribution level of culture and tourism integration efficiency. Low integration efficiency regions include three cities, Dezhou, Liaocheng and Heze, which are all distributed in the west. Among them, Heze has the lowest integration efficiency, which is 0.139. In 2015, although there were no high integration efficiency regions, the spatial differentiation characteristics were the same as in 2010. The areas with high integration efficiency have been reduced to three cities, all of which are located in the eastern region. The highest integration efficiency is 0.743 in Qingdao; the regions with medium integration efficiency have increased to ten cities, accounting for 62.50% of the total. Regions with low integration efficiency include Rizhao, Liaocheng and Heze, among which Heze still has the lowest integration efficiency at 0.169, but an increase of 21.58% compared with 2010. In 2019, the culture and tourism integration efficiency generally showed a spatial distribution pattern of "high in the central and low in the north and south". The only region with high integration efficiency is Qingdao, and its integration efficiency has continued to increase from 0.743 in 2015 to 0.783 in 2019. The regions with relatively high integration efficiency have increased, all distributed in Jiaodong and central regions. The medium integration efficiency areas are still maintained in ten cities, except for Rizhao and Jining, all of which are located in southwestern and northwestern regions.

Based on the analysis of 2010, 2015 and 2019, the culture and tourism integration efficiency in Dezhou, Liaocheng, Heze and Jining has changed from low to high distribution grades, and the quality of culture and tourism integration efficiency has continued to improve. Due to the relatively backward economy and incomplete infrastructure construction in these regions, culture and tourism integration efficiency is low. However, benefiting from the "Yellow River Cultural Tourism Belt" initiative in recent years, the government has increased the construction of public cultural tourism service system, accelerated economic development, and created good conditions for culture and tourism integration efficiency. In addition, the beautiful natural scenery and characteristic regional culture in these areas have also accelerated culture and tourism integration efficiency. The distribution level of Tai’an, Linyi and Yantai has developed from high to low distribution grades, and the culture and tourism integration efficiency has deteriorated. These regions have developed economy and abundant cultural tourism resources, and culture and tourism integration efficiency has been at a high level for many years. In recent years, the problems of backward concept of cultural tourism consumption, lagging development of cultural tourism consumption market, shortage of human resources and technology have become more and more obvious. The level of culture and tourism integration efficiency in other regions has not changed.

### Spatial agglomeration evolution characteristics

The spatial agglomeration phenomenon and characteristics of culture and tourism integration efficiency in Shandong Province were revealed via exploratory spatial data analysis (ESDA). By taking the K-nearest neighbor matrix as the weight matrix of spatial relation, we calculated the global Moran’s indexes of culture and tourism integration efficiency in Shandong Province from 2010 to 2019, as listed in Table 3. According to the calculated Z-values and p-values, only the results in 2013 and 2016 did not reach statistical significance, and the results in the rest of the years reached the 5% significance level. Moreover, the global Moran’s indexes in different years were positive, suggesting a positive spatial correlation on the whole. From the change of the global Moran’s index, it always fluctuated between 0.152 and 0.250, indicating that the spatial agglomeration of culture and tourism integration efficiency in Shandong Province was relatively stable during the research period, with obvious regional differences and obvious path dependence characteristics. Tobler’s First Law of Geography points out that "Everything is related to Everything else, but near things are more related to each other". The 30 samples determined by spatial statistical analysis are only recommended values, but not definitive values [48]. Since there are only 16 cities in Shandong Province, Student’s T test was further used for supplementary verification to avoid the credibility and persuasiveness of the results being questioned [49]. The results show that the P values of the output results reach a significant level except for individual years. Therefore, the spatial statistical analysis is suitable for this paper.

**Table 3:** Global Moran’s indexes of culture and tourism integration efficiency in Shandong Province.

Year	2010	2011	2012	2013	2014	2015	2016	2017	2018	2019
Moran’s I	0.213	0.152	0.248	0.101	0.215	0.284	0.109	0.197	0.250	0.184
Z-score	1.674	1.330	1.960	1.007	2.284	2.152	1.039	1.588	1.892	1.993
p-value	0.047	0.072	0.025	0.157	0.022	0.016	0.149	0.056	0.029	0.045

In order to further analyze the local spatial characteristics of culture and tourism integration efficiency distribution, we drawn and summarized the local Moran indexes scatter charts for 2010, 2015 and 2019 (as shown in Table 4). The local Moran indexes scatter charts were divided into four quadrants: The first quadrant represents H (High)-H (High) agglomeration regions, i.e., the culture and tourism integration efficiency in the city itself and neighboring cities are both high. The second quadrant represents H (High)-L (Low) agglomeration regions, i.e., the culture and tourism integration efficiency in the city itself is high, and the neighboring city is low. The third quadrant represents L (Low)-H (High) agglomeration regions, i.e., the culture and tourism integration efficiency in the city itself is low, and the neighboring city is high. The fourth quadrant represents (Low)-L (Low) agglomeration regions, i.e., the culture and tourism integration efficiency in the city itself and neighboring city are low. Specifically, the H-H agglomeration regions are mainly concentrated in the Jiaodong Peninsula, and the number of cities has expanded with time. Moreover, the agglomeration effect has become more significant, forming a growth pole for the integration efficiency of regional culture and tourism. The L-L agglomeration regions are mainly distributed in southwestern and northwestern Shandong Province, showing a "U" shape development pattern in the number of cities, indicating that the culture and tourism integration efficiency has a certain spatial locking. Combined with the spatial characteristics of H-H and L-L agglomeration regions, to a certain extent, it can be considered that the spatial spillover effect of culture and tourism integration efficiency in Shandong Province is mainly manifested in the following aspects: First, the spatial spillover effect between cities with the same level of integration efficiency is positive; Secondly, the leading and radiating effect of cities with high integration efficiency on cities with low integration efficiency is not obvious. On the contrary, it shows the "siphon effect" of cities with high integration efficiency on capital, labor, technology and other resources of cultural tourism industry in cities with low integration efficiency.

**Table 4:** Summary table of local Moran’s indexes scatter charts of culture and tourism integration efficiency in Shandong Province.

Quadrant	2010	2015	2019
First quadrant(H-H)	Qingdao, Yantai, Weifang, Weihai	Qingdao, Zibo, Yantai, Weifang, Weihai	Qingdao,Yantai, Weifang, Tai’an, Weihai
Second quadrant(L-H)	Dongying, Binzhou, Rizhao	Dongying, Tai’an, Binzhou, Rizhao	Binzhou, Rizhao
Third quadrant(L-L)	Jinan, Zaozhuang, Jining, Dezhou Liaocheng, Heze	Zaozhuang, Dezhou,Liaocheng, Heze	Zibo, Zaozhuang, Dongying, Dezhou, Liaocheng, Heze
Fourth quadrant(H-L)	Zibo, Tai’an, Linyi	Jinan, Jining, Linyi	Jinan, Jining, Linyi

### Analysis of influencing factors of culture and tourism integration efficiency

#### Model selection

Before model establishment, we performed a stationarity test on the above all variables so as to avoid the spurious regression. From the unit root test results, in the level value of Levin-Lin-Chu (LLC) and ADF-Fisher (augmented dickey-fuller) test methods, all variables except *lnINFO* rejected the null Hypothesis at a significance level of 1%. In order to further test the stationarity of the panel dataset, it was necessary to carry out the first-order difference unit root test on the all variables. The final test results showed that in the LLC test and the ADF-Fisher test, all variables rejected the null Hypothesis at a significance level of 1%, indicating that the panel dataset is a first-order single integer stable sequence data. Moreover, the co-integration test analysis could be performed on the established panel dataset. In the case of homogeneous panel data, the Kao test and the Pedroni test all rejected the null Hypothesis at the 1% significance level, indicating that there is a long-term stable equilibrium relationship between *FE* and the influencing factors. From the calculation results in Table 3, it can be seen that the spatial correlation of *FE* passed the 1% significance level test and showing that the spatial econometric model should be adopted. Further, in this paper, the method of Hausman test was used to select the model form by calculating the *P* statistic value. From the calculation results, it can be known that the *P* statistic value is 0.000, indicating that the spatial econometric model with fixed-effect could better explain the relationship between *FE* and the influencing factors. The statistics of the Wald (SAE) and LR (SEM) are 55.35 and 33.98 respectively, and both reject the hypothesis of *θ =* 0 and *θ = -βρ*. Obviously, the SDM model had a high goodness of fit. In order to improve the robustness of the regression results, the SDM model (Model 1) and the DSDM model (Model 2-Model4) were tested respectively, as shown in Table 5. Compared with the SDM model, the R^2^ of the DSDM model was 0.613, indicating that the goodness of fit of DSDM model was relatively high. There is more fitting degree when the time lag term of dependent variable and the space-time lag term are introduced into DSDM. So Model (4) was selected as the optimal estimation model in this paper. Correlation regression results were estimated by the xsmle command in Stata^TM^ Version 16.0 software.

**Table 5:** Regression results of SDM and DSDM models.

Variable	Model(1)	Model(2)	Model(3)	Model(4)
*L*.*lnFE*		0.021(0.27)***		0.029(0.36)**
*L*.W*lnFE			-0.315(-1.13)**	-0.318(-1.15)***
*lnECON*	0.137(3.34)***	0.227(4.29)***	0.231(4.78)***	0.217(4.15)***
*lnHC*	-0.058(-1.01)	-0.036(-1.63)	-0.034(-1.59)	-0.035(-1.60)
*lnPE*	0.004(0.14)	0.309(2.87)*	0.022(2.59)*	0.022(-2.60)*
*lnINFO*	0.124(2.85)***	0.111(1.22)***	0.120(1.32)***	0.120(1.32)***
*lnTC*	0.116(2.16)**	0.354(1.71)	0.370(1.79)*	0.365(1.76)*
*OPEN*	0.024(0.50)	0.046(0.91)**	0.046(0.91)**	0.047(0.94)**
W*lnECON	0.110(1.57)	0.076(0.54)	0.106(0.74)	0.107(0.76)*
W*lnHC	-0.009(-0.10)	-0.135(-0.86)	-0.019(-0.11)	-0.023(-0.12)
W*lnPE	-0.077(-1.43)	-0.375(-3.04)**	-0.345(-2.75)**	-0.351(-2.78)**
W*lnINFO	-0.104(-5.12)***	-0.153(-4.34)**	-0.161(-4.65)**	-0.161(-4.65)**
W*lnTC	-0.008(-0.07)	-0.082(-0.09)	-0.014(-0.01)	-0.037(-0.04)
W*OPEN	0.243(2.76)***	0.359(3.29)***	0.204(3.66)***	0.204(3.66)***
Spatial rho	0.603(8.06)***	0.496(6.10)***	0.485(4.97)***	0.553(5.56)***
Time-fixed effect	Yes	Yes	Yes	Yes
Individual-fixed effect	Yes	Yes	Yes	Yes
*R* ^*2*^	0.609	0.667	0.613	0.687

Values in parentheses are *t* values.

*** indicate the factors were significant at a level of 0.01

** indicate the factors were significant at a level of 0.05, and

* indicate the factors were significant at a level of 0.1.

#### Model estimation results

As DSDM model displays at the fourth column in Table 5, the spatial autocorrelation coefficient *ρ* of culture and tourism integration efficiency is 0.553 and significantly positive at 1%, which is consistent with the test results of the Moran’s indexes. It proved that the culture and tourism integration efficiency in Shandong Province has positive spatial spillover effect and indicating that the improvement of culture and tourism integration efficiency in local cities will lead to the improvement of culture and tourism integration efficiency in neighboring cities. The coefficient of *L*.*lnFE* is significantly positive at the level of 5%, indicating that the culture and tourism integration efficiency in Shandong Province has a strong path dependence in the time dimension. Under the promotion of cross-border integration of cultural tourism, a region with high level of culture and tourism integration efficiency in prior-period has a positive influence on current-period culture and tourism integration efficiency improvement of itself, showing a significant "superposition effect". The coefficient of *L*.W*lnFE is -0.5271 and significantly negative at 10%, suggesting that cities with higher culture and tourism integration efficiency in prior-period, it will go against the current-period culture and tourism integration efficiency improvement of its neighboring cities. One of the possible reasons is cultural tourism capital, labor, technology, and other resources have a "siphon effect" at city level. Cities with a high level of culture and tourism integration efficiency will attract superior resources from its neighboring cities, giving rising to a negative impact on the future culture and tourism integration efficiency improvement of the adjacent cities. From the absolute value of the three coefficients, the spatial spillover effect of culture and tourism integration efficiency is dominant.

For the spatial econometric model, owing to the existence of spatial dependence, the coefficient of independent variables was no longer appropriate for explaining the influence of independent variables on dependent variables [50]. However, the DSDM models enable us to empirically study the time effects and space-time effects in the short-term and long-term. The influence of variables could be measured in the reference city (direct effect) and in the neighboring cities (indirect effect). Table 6 shows the short-term and long-term direct effects, indirect effects of the DSDM model.

**Table 6:** Direct and indirect effects of the factors influencing culture and tourism integration efficiency.

Variable	Short-term effect		Long-term effect	
	Direct effect	Indirect effect	Direct effect	Indirect effect
*lnECON*	0.046(0.58)**	0.086(0.74)**	0.048(0.53)*	0.091(0.65)**
*lnHC*	0.008(0.14)***	0.034(0.31)	0.009(0.12)***	0.286(0.30)
*lnPE*	-0.066(1.88)*	-0.217(2.97)***	-0.078(1.69)*	-0.282(2.86)***
*lnINFO*	0.100(1.16)	0.030(0.33)	0.113(1.14)**	0.039(0.44)**
*lnTC*	0.141(0.64)*	-1.150(-2.51)**	1.172(0.82)**	-1.327(-2.62)***
*OD*	0.058(0.95)	0.044(0.37)	0.065(0.91)**	0.319(0.29)***

Values in parentheses are *t* values.

As shown in Table 6, whether direct effect or indirect effect, the long-term effect of each independent variable is greater than the short-term effect (absolute value), which indicates that each independent variable can has a profound long-term impact on the culture and tourism integration efficiency and has a cumulative effect.

The direct and indirect effects of the level of economic development are significantly positive in the short and long term, which confirms that the rapid economic development in local cites can not only have a positive impact on local culture and tourism integration efficiency but also have a positive impact on the culture and tourism integration efficiency of its surrounding areas. On the one hand, the level of economic development is the base of the development of regional cultural tourism industry. The higher the level of economic development, the greater the motivation and potential of local residents’demand for cultural tourism. And the increase of demand will further promote local cultural tourism investment and the construction of public cultural tourism facilities, forcing the improvement of culture and tourism integration efficiency. On the other hand, the higher the level of economic development in a region, the stronger the level of capital, science and technology. The more likely it is to export to the neighboring regions, thus forming an effective radiation to improve the culture and tourism integration efficiency in neighboring cities.

The improvement of human capital level has a positive impact on the culture and tourism integration efficiency in local cities in the short and long term, but not on the neighboring cities. It means that the accumulation of human capital in local cities has produced obvious economies of scale effects on the improvement of factor productivity and the adjustment of industrial structure of cultural tourism industry. However, the "proximity" trend of talent flow in Shandong Province may weaken the scale effect of human capital accumulation in other cities, thus not significantly improving the culture and tourism integration efficiency.

In the short and long term, the impact of policy environment on culture and tourism integration efficiency shows significant negative direct effect and significant negative indirect effect. It indicates that when other conditions remain unchanged, the industrial environment has a negative impact on local culture and tourism integration efficiency as well as its surrounding cites. For one thing, the industrial policy has led to the distortion of the allocation of cultural tourism elements to a certain extent, which is not conducive to the improvement of culture and tourism integration efficiency in local cities. For another, the distortion of the allocation of cultural tourism elements caused by industrial policies has resulted in a situation of "a mutual stake" at the spatial level, and that the adverse impact of local urban industrial policies on culture and tourism integration efficiency has not brought warning to neighboring cities.

The short-term direct and indirect effects of the level of informatization development and the openness degree do not pass the significance test. From the perspective of long-term direct and indirect effect, coefficients of the level of informatization development and the openness degree are significantly positive, indicating that the level of informatization development and the openness degree have only a long-term effect on the culture and tourism integration efficiency, but not a short-term effect. At present, the level of information development in Shandong Province has not had a significant impact on the culture and tourism integration efficiency in various cities in the short term. But with the continuous extension and expansion of the Internet, big data, cloud computing, artificial intelligence, AR/VR/MR and other applications in the field of cultural tourism, it will further accelerate the cultural tourism industry chain to high-end, and promote the culture and tourism integration efficiency in the long run. The openness degree for the development of cultural tourism industry in Shandong Province is not high. In 2019, the total amount of foreign capital utilized by the cultural tourism industry accounted for only 4% of the total amount of foreign capital utilized by the tertiary industry, and the flow of foreign capital to the industry was not balanced. More foreign capital is invested in catering and accommodation industries. In the short-term, it is impossible to play a scale economy effect and technology spillover effect on the culture and tourism integration efficiency between local and neighboring cities. However, with the continuous increase of the total amount of foreign capital utilization and the diversification of the source structure of foreign capital in Shandong’s cultural tourism industry, foreign capital agglomeration gives its effection. It will not only promote the culture and tourism integration efficiency by improving the clean production technology and intensive production level of local cultural tourism industry, but also improve the culture and tourism integration efficiency by strengthening the construction of green development system of cultural tourism in neighboring cities through demonstration effect.

The direct effect of traffic condition is significantly positive and the indirect effect is significantly negative, both in the long and short term. On the one hand, it means that good urban traffic condition can eliminate the spatial barrier of the development of cultural tourism industry, accelerate the agglomeration and diffusion effects of the elements of the cultural tourism industry, and expand the demand scale of cultural tourist market. On the other hand, it shows that the better the local urban traffic condition is, the more centripetal effect will be exerted on the elements of cultural tourism industry in neighboring cities, which will promote the development of culture and tourism integration efficiency in local cities to tend to the growth pole mode. Thus it can aggravate the regional imbalance of culture and tourism integration efficiency.

#### Robustness tests

In this paper, the K-nearest neighbor weight matrix is used in the spatial panel econometric analysis. In the robustness test, the reciprocal of the city’s straight-line distance is selected as the spatial weight matrix for estimation. From the results, the impact of the change in the selection of the spatial weight matrix is not obvious, and the sign and significance of the independent variable coefficients are basically consistent with the regression results above, which confirms the robustness of the empirical results.

## Conclusions and discussion

### Conclusions

Based on the the inherent requirements of high-quality development, this paper has analyzed the culture and tourism integration efficiency of 16 cities in Shandong Province, China from 2010 to 2019. We have systematically analyzed the spatio-temporal evolution characteristics of Shandong’s culture and tourism integration efficiency. We have also analyzed the influencing factors on culture and tourism integration efficiency. This research leads to several key outcomes.

Over the period from 2010 to 2019, the overall time evolution trend of culture and tourism integration efficiency can be divided into three stages: rapid growth, rapid decline and stable rise period. From 2010 to 2011, it was a period of rapid growth. The culture and tourism integration efficiency increased from 0.446 to 0.569, an increase of 27.57%. It is mainly due to the development strategy of expanding domestic demand and stimulating consumption. From 2011 to 2014 is a period of rapid decline, with large fluctuations in the decline, and the "instability" characteristics of the curve have become increasingly prominent. It is mainly affected by the unreasonable allocation of resources in the cultural tourism industry. From 2014 to 2019 is a period of stable climbing, with an average annual increase of 5.38% in culture and tourism integration efficiency, and the growth rate is gradually stable. This is mainly owing to the influence of cultural tourism industry system reform and management innovation.

During the study period, the spatial pattern of culture and tourism integration efficiency has changed from "high in the east and low in the west" to "high in the middle and low in the north and south". The culture and tourism integration efficiency cities in Shandong Province shows a significant positive spatial correlation. The state of spatial agglomeration is relatively stable, with obvious regional differences and obvious path dependence characteristics. Specifically, the high-efficiency agglomeration regions (H-H) of culture and tourism integration are mainly concentrated in the Jiaodong Peninsula, and the number of cities has expanded with time. Moreover, the agglomeration effect has become more significant, forming a growth pole for the integration efficiency of regional culture and tourism. The low-efficiency agglomeration regions (L-L) of culture and tourism integration are mainly distributed in southwestern and northwestern Shandong Province, showing a "U" shape development pattern in the number of cities, indicating that the culture and tourism integration efficiency has a certain spatial locking.

The culture and tourism integration efficiency shows obvious positive spatial spillover effect, "superposition effect" and "siphon effect", and the spatial spillover effect is dominant. On account of different influencing strengths and directions of various influencing factors among different regions, the culture and tourism integration efficiency shows obvious spatial-temporal characteristics. The level of economic development significantly promotes the culture and tourism integration efficiency in local and neighboring cities in the short and long term, while policy environment has a significant negative impact. Traffic conditions and human capital only promote the culture and tourism integration efficiency in local cities. Information development level and openness degree only have a long-term effect on the culture and tourism integration efficiency, but not a short-term effect.

### Policy suggestions

Combining the above empirical analysis results, in order to improve the culture and tourism integration efficiency in Shandong Province, promote the high-quality development of cultural tourism industry and the construction of a strong cultural tourism province, we can make the following policy suggestions.

Firstly, there was a strong spatial correlation of the culture and tourism integration efficiency of 16 cities in Shandong Province. We should strive to overcome the administrative barrier and perfect the cultural tourism competition-cooperation mechanism among cities. Accordingly, the driving role of Jiaodong Peninsula region can be brought into fully play. The maximization of positive spillover effect of economic development, opening up and information resources on the central, southwestern and northwestern regions of Shandong Province can be promoted so as to further narrow the regional gap and achieve high-quality and balanced development of Shandong′s cultural and tourism integration efficiency. At the same time, the central, southwestern and northwestern regions of Shandong should actively stimulate their own new kinetic energy for the development of cultural tourism, thereby highlighting the development mode and path of the cultural tourism industry with intensive elements.

Secondly, the government’s intervention in the cultural tourism market is suppose to be regulated. The decisive role of the market in the allocation of resources and elements of the cultural tourism industry, and the influence of the government in the development of the cultural tourism industry should be brought into full play. Such as the guidance and regulation of the allocation of cultural tourism resources, the construction of preferential system of investment and financing and fiscal and taxation policies, etc. Thereby promoting the policy environment to play its due role in improving the culture and tourism integration efficiency.

Thirdly, we should build a long-term guarantee and correlation mechanism for the cross-regional flow of human capital in the cultural tourism industry, and form complementary advantages and linkage development in space. Meanwhile, we should strongly advocate the role of local human capital in improving the culture and tourism integration efficiency in neighboring cities. According to this, the scale effect and external effect of human capital accumulation can be fully reflected in a larger space.

Finally, we should implement the specific requirements of the "14th Five-Year Plan for Comprehensive Transportation Development in Shandong Province", accelerate the realization of the interaction and interconnection of transportation infrastructures and fully improve the overall traffic accessibility in regions with low culture and tourism integration efficiency. Thereby reducing the flow cost of resource elements, better accepting the spillover effect of transportation infrastructure in the Jiaodong Peninsula region and accelerating the formation of a novel uniform cultural tourism pattern.

### Limitations and future research

Under the background of high-quality development, gaining in-depth knowledge of spatio-temporal evolution characteristics and revealing the influencing factors can contribute to strengthening the overall integration of cultural tourism resources in Shandong Province and optimizing the spatial layout of cultural tourism. This study adopted the benevolent DEA cross-efficiency model to measure the culture and tourism integration efficiency. Although the final efficiency value of each DMU takes into account the optimal weights of all DMUs, the optimal weights selected by any DMU is only the most favorable to itself, which is not conducive to the peer-appraisal efficiency value of other DMUs. Therefore, it can not fully reflect the operation and management conditions of regional cultural tourism. At the same time, due to the availability of urban unit data in Shandong Province, the selected evaluation indicator system and related influencing factors of culture and tourism integration efficiency do not involve indicators, which are difficult to quantify or have poor continuity. To some extent, it affects accurate description of the performance of cultural tourism industry in this research. Consequently, in future research, more indicators should be absorbed into reveal the properties and rules during the development of cultural tourism industry, so as to further promote the allocation of cultural tourism resources to maximize benefits and optimize efficiency.

## Supporting information

S1 Data (XLSX)

S2 Data (XLSX)
